# 

**Published:** 1855-03

**Authors:** 


### RECEIPTS FROM JAN. 27 UP TO FEB. 27, 1855.

ILLINOIS.

| | |
|---|---|
| Dr E S Cooper | $4 00 |
| J W H Davis | 2 00 |
| A L McArthur | 2 00 |
| C G Strobecker | 2 00 |
| T R May | 2 00 |
| S Cummings | 2 00 |
| J A Faris | 2 00 |
| Dr W E Wilson | $2 00 |
| L A Engle | 2 00 |
| R S Halleek | 2 00 |
| J C McMurtry | 2 00 |
| Elias Wenger | 2 00 |
| L Hale | 1 00 |
| J W Trabue | 2 00 |

INDIANA

| | |
|---|---|
| Drs Constant and Walker | 5 00 |
| Dr J W Newlin | 9 00 |
| M R Chadwick | 2 00 |
| Dr J Newton | $2 00 |
| B F Hutson | 2 00 |
| Dr R M Gilkeson | $2 00 |
| Wm Manson | 2 00 |

WISCONSIN.

| | |
|---|---|
| Dr H Warne - - | $2 00 |
| Edward Hopkins - - | 2 00 |

MICHIGAN.

| | |
|---|---|
| Dr James M Higby - - | 2 00 |
| A De Armend - - | 2 00 |

| | |
|---|---|
| Dr G N B Elliott, Iowa. - | 2 00 |
| G F Beekner, Missouri | 2 00 |
| R L Brown, Pennsylvania. | 2 00 |

